# Attitudes Toward Multilingualism in Luxembourg. A Comparative Analysis of Online News Comments and Crowdsourced Questionnaire Data

**DOI:** 10.3389/frai.2020.536086

**Published:** 2020-10-22

**Authors:** Christoph Purschke

**Affiliations:** Department of Humanities, Institute for Luxembourgish Language and Literature, University of Luxembourg, Esch-sur-Alzette, Luxembourg

**Keywords:** computational sociolinguistics, attitudes, crowdsourcing, low-resource languages, Luxembourgish, multilingualism, orthographic normalization, representation learning

## Abstract

Attitudes are a fundamental characteristic of human activity. Their main function is the situational assessment of phenomena in practice to maintain action ability and to provide orientation in social interaction. In sociolinguistics, research into attitudes toward varieties and their speakers is a central component of the analysis of linguistic and cultural dynamics. In recent years, computational linguistics has also shown an increased interest in the social conditionality of language. To date, such approaches have lacked a linguistically based theory of attitudes, which, for example, enables an exact terminological differentiation between publicly taken *stances* and the assumed underlying *attitudes*. Against this backdrop, the present study contributes to the connection of sociolinguistic and computational linguistic approaches to the analysis of language attitudes. We model a free text corpus of user comments from the RTL.lu news platform using representation learning (*Word2Vec*). In the aggregated data, we look for contextual similarities between vector representations of words that provide evidence of stances toward multilingualism in Luxembourg. We then contrast this data with the results of a quantitative attitudes study, which was carried out as part of the crowdsourcing project “Schnëssen.” The combination of the different datasets enables the reconstruction of socially pertinent attitudes represented in public discourse. The results demonstrate the central importance of attitudes toward the different languages in Luxembourg for the cultural self-understanding of the population. We also introduce a tool for the automatic orthographic correction of Luxembourgish texts (*spellux*). In view of the ongoing standardization of Luxembourgish and a lack of rule knowledge in the population, orthographic variation—among other factors like code-switching or regional dialects—poses a great challenge for the automatic processing of text data. The correction tool enables the orthographic normalization of Luxembourgish texts and with that a consolidation of the vocabulary for the training of word embedding models.

## Introduction

Attitudes toward language and other cultural phenomena are one of the basic characteristics of social practice. They play a central role for the way people use, perceive, and evaluate language. For example, the assessment of a social style or regional variety (e.g., as opposed to the standard variety) in a specific situation has an impact on behavior in competitive situations (Heblich et al., 2015). The same holds true for how people perceive other people in terms of character traits or other aspects of social interaction (Kristiansen, 2009). Attitudes arise in practice in the form of “relevance-driven targeting and evaluation routines […] that sediment in an individual's stock of knowledge and are situationally (re)constructed in interaction” (Purschke, 2015, p. 49). Attitudes are therefore routinized *judgments* about phenomena in everyday life, which can become apparent in interaction in the form of *stances* (Jaffe, 2013), that is, in speech acts or other types of action. However, there is no demonstrable direct link between a person's attitudes and their actions (Soukup, 2012). The reason for this lies in the diverse implicit and explicit, self-related and social *norms* that determine social interaction, and, therefore, the emergence, structuring, and externalization of attitudes. For example, not every attitude is *socially appropriate* in every situation, such as politically controversial opinions when talking to a superior at work. In addition, not all attitudes are equally *cognitively accessible* and *consciously controllable* with regard to their appearance in and relevance for action (Pharao and Kristiansen, 2019). As a consequence, we have to take into consideration different aspects of the *cognitive organization, social embedding*, and *practical functions* of attitudes, for example, the complex relation between the long-term stability of many attitudes (e.g., prejudice against certain dialects; Preston, 2015), their general changeability through new experiences (e.g., through direct contact with speakers of a stigmatized variety; Giles and Marlow, 2011), and their situational expression in concrete interactions (e.g., the use of dialectal features as stance markers in chat communication; Tophinke and Ziegler, 2014).

Research on attitudes dates back to the early days of psychology and has been a topic of long-standing tradition in the humanities and social sciences. In sociolinguistics, attitudes have been examined with a wide range of methodological approaches and against the backdrop of different theoretical frameworks. Albarracín and Johnson (2018) provide a good overview about the development of the field. Regardless of methodological and theoretical discussions about how to describe and survey attitudes best, it has been shown that and to what extent attitudes are important for the practical organization of social interaction. For example, in the German speaking area, the perception and evaluation of linguistic variation is closely related to the overall dynamics of regional dialects, and this connection derives directly from the sociocultural orientation of the language users (Purschke, 2018). In addition, people's attitudes toward language in general and the different varieties present in a speech community substantially influence their migration behavior (Lameli et al., 2015).

At the same time, this close connection between language use and language evaluation poses one of the biggest challenges to the computational processing and modeling of language in computational linguistics (Hovy, 2018). Basic traits of language practice, such as social meaning, irony, mimicking citation, and other forms of stylization cannot reliably be detected and processed by algorithms (e.g., in tasks like sentiment analysis, machine translation, or chat bots). Furthermore, models and algorithms work best for standardized datasets in high-resource languages and seem to reproduce aspects of demographic and social bias in automated processing (Garimella et al., 2019). As a consequence, the applicability and appropriateness of many NLP applications for everyday language is still limited, despite the great advances in computer science and AI research (Bender and Koller, 2020). In recent years, there has been a new trend in the NLP community that is increasingly concerned with language as a social phenomenon and that tries to incorporate sociolinguistic knowledge into the analysis of data and the development of new tools and models (Broadwell et al., 2013; Eisenstein, 2015; Nguyen, 2017; Purschke and Hovy, 2019).

This article is committed to the same goal. The aim of the text is to reconstruct language attitudes toward multilingualism in Luxembourg with the help of different data types. On the one hand, we aggregate stances toward language and multilingualism in free text data and evaluate them using computational linguistic methods. We then compare the data with the results of a sociolinguistic questionnaire survey that was carried out with the help of a mobile crowdsourcing application. A comparison of the different data types shows that attitudes can be successfully reconstructed from free text data and that the patterns found reflect the attitudes of people toward multilingualism in Luxembourg as well as certain aspects of public discourse. In terms of methodology, the text thus makes a contribution to the field of computational sociolinguistics by trying to systematically relate computational linguistic and sociolinguistic approaches in analysis. From a theoretical point of view, the article provides proof of the importance of *contextual knowledge* for the organization of social practice, with a special regard to the role of attitudes as *situated evaluation routines*. Furthermore, the article contributes to the development of computational linguistic resources for Luxembourgish as a low-resource language, that is, the automatic normalization of orthographic and regional variation in text data.

## Multilingual Luxembourg

The sociolinguistic setting in Luxembourg is comparably complex. It has developed as a result of a fickle history in contact with neighboring cultures (especially France and Germany). In addition, socio-economic migration, the country's specialization in the private financial industry, and the presence of several European institutions play an important role in the emergence and dynamism of the current language regime. With a total population of 613,000, the Grand Duchy has a very high proportion of foreign residents of 47.5%. In addition, there are 192,000 cross-border commuters coming in from Germany, France, and Belgium every day (STATEC, 2019). The country has three official languages, the national language Luxembourgish, and French and German as administrative languages. Luxembourgish multilingualism is also characterized by strong minority languages (Portuguese, Italian) and an increasing presence of English. Language use and the social embedding of the different languages in Luxembourg are organized on a domain-specific basis (Erhart and Fehlen, 2011). For example, French serves as the language of legislation and jurisdiction, but debates in Parliament take place in Luxembourgish. The print media are traditionally dominated by German (and to a limited extent French), while radio and national television broadcast largely in Luxembourgish. German is the language of alphabetization, but the school system as a whole is also designed to promote multilingualism, with a strong copresence of French. Luxembourgish is the language of everyday communication among Luxembourgers and has undergone processes societal and political *Ausbau* in the past 15 years (Gilles, 2019), which have resulted, among other things, in a new law promoting Luxembourgish in 2018, by means of which its societal anchoring is to be strengthened. The language has developed into a written variety that is suitable for all communicative occasions, from informal communication in social media to public inscriptions and formal letters, and the official orthography has been consolidated and modernized in 2019. At the same time, the majority of the population does not have an in-depth knowledge of the official spelling rules, because Luxembourgish is not an integral part of the school curriculum.

Given its sociocultural diversity and strong demographic dynamics (the population has grown by 39.7% since 2001; STATEC, 2019), the language regime is currently on the move. While Luxembourgish is increasingly present in all social domains, the role of German as a bridge language (traditionally seen as “written Luxembourgish”; Kloss, 1952) is clearly decreasing. At the same time, the importance of French is increasing, above all because of the high proportion of foreign employees in the private sector. Additionally, the social presence of English is increasing due to its growing importance for tourism, economy, and pop culture. While French traditionally plays the role of a cultural prestige language, the young generation in particular shows a clear preference for English (and indirectly German due to its close relationship with Luxembourgish). Multilingualism and especially the societal role of Luxembourgish have been frequent topics in public debates in recent years (Horner and Weber, 2008; Garcia, 2014). Following a referendum on the right to vote for foreigners in 2015 and an increasing politicization of language in public discourse and political action, the discussion about the languages of the country has developed into a “replacement discourse.” In this context, languages serve as a proxy for societal disputes, for example, the demographic development, rising living costs, and democratic legitimation of politics. Many of these topics can also be found in discussions on social media (especially Facebook) and in the user comments of the RTL.lu news platform (*Example 1*).

**Example 1: Language-related comment from the RTL data set**
*Et soll endlech klip % klor gesetzlich verankert gin das jus nach L hei emgangssproch ass, d.h wen well hei schaffen op brout verkafen oder deck plaz op da bank MUSS L kennen. Dat muss dach meiglich sin* [2016-02-21].
**Translation:** It should finally be anchored in the law that Luxembourgish is the only colloquial language here, which means that anyone who wants to work here, whether selling bread or a fat job in the bank, must be able to speak Luxembourgish. That must be possible.

In this example, the author takes a clear stance on the language regime by demanding Luxembourgish as the only colloquial language for the country. They combine this with a demand for linguistic integration from foreign workers. In addition to the close connection between linguistic and societal issues in public discourse, the comment also illustrates some of the challenges in dealing with Luxembourgish text data: The text contains many spelling mistakes (e.g., *jus* instead of *just* “just, only,” *emgangsproch* instead of Ë*mgangssprooch* “colloquial language”), irregular use of capitalization and punctuation, abbreviations like *L* for *Lëtzebuergesch* “Luxembourgish,” and colloquial expressions. This variability poses a particular challenge for automated text processing, especially because of the large amount of orthographic variation.

Against the backdrop of the complex and dynamic Luxembourg multilingualism, the aim of the present study is to examine the attitudes of the population toward multilingualism and the role of Luxembourgish in particular. On the one hand, the analysis is based on user comments from the RTL.lu news platform, on the other hand, answers from a sociolinguistic questionnaire survey on attitudes toward multilingualism are taken into consideration.

## Data and Methods

In the following section, the different data sources are discussed. This involves the respective characteristics of the data, but also their preparation and modeling for the subsequent analysis. First, we present the user comments from RTL.lu. In this context, we discuss the particular challenges when working with Luxembourgish text data that require a special preprocessing workflow. In a second step, we discuss the questionnaire data. Since these data stem from a crowdsourcing project, certain preprocessing steps are also necessary in this case.

### Mining Attitudes From RTL.lu User Comments

#### Dataset

The data for the computational linguistic analysis stem from the RTL.lu news platform. The RTL media group is the largest news provider in the country and has television and radio programs as well as a widely used online news portal. The platform has existed since 2008 and is the only news offering to date that is entirely in Luxembourgish. As part of a project to develop semantic annotation algorithms for Luxembourgish text data at the University of Luxembourg (“STRIPS” project; Gierschek et al., 2019), RTL has made all the articles published on the platform as well as the associated user comments available for research. The project primarily uses the data to measure sentiment in user comments. In addition, the data can also be used for the investigation of orthographic variation (temporal development of correctness and individual norm accommodation) or for discourse analytical questions, for example, the reconstruction of language attitudes.

The dataset comprises a total of 179,298 news articles and 585,358 user comments from the period between 2008 and 2018. All comments are anonymous and, in addition to a time stamp, contain information about the article to which they refer. Thematically, the corpus covers the entire range of topics offered on the media platform: national and international news, topics from society, culture, and science, sports, local journalism, but also reader contests or reports. The majority of the texts are written in Luxembourgish. While the news articles are largely spelled correctly orthographically, the user comments show diverse sources of linguistic variation:

- *correctness:* Since the development of Luxembourgish as a written variety has taken place over the past 15 to 20 years and its standardization has not yet been completed, the early contributions tend to show a greater orthographic variation than more recent contributions, especially with regard to their correctness. In view of the lack of social anchoring of the official rules in the population, however, the recent contributions are also very variable orthographically.
- *formality:* The comments express a range of textual formality, from some early comments similar to letters (with a salutation and signature) to informal texts typical for online communication that are conceptually largely based on oral language.
- *mediality:* The texts show the expected range of medium-specific writing resources that are typical for digital writing. This includes variable use of upper- and lower-case letters, the use of emoji and acronyms, irregular punctuation, or onomatopoetic writing to express emphasis.
- *regionality:* In addition to orthographic variation, the texts are also characterized by regional variation. Although extensive processes of dialect leveling have already taken place in Luxembourgish, there are still diverse traces of regional spellings in the texts, e.g., forms like *wuar* or *woar* for *war* “was”.
- *multilingualism:* While the majority of the contributions is written in Luxembourgish, the multilingual competence of the writers results in many texts that contain elements of code-switching into German, French, or English. In addition, there is the characteristic of Luxembourgish as a “hybrid” language, that is structurally close to German and at the same time has integrated many elements from French.

These characteristics of online writing are not exclusive to Luxembourgish. In fact, we find some of them (correctness, regionality) in many smaller languages that have not been (fully) standardized, while others (formality, mediality) are typical for (the development of) online writing in general, as is code-switching in multilingual communities. However, the combination of the different characteristics, combined with the comparatively good availability of machine-readable data, represents a special feature of Luxemburgish as a research topic. Additionally, the Luxembourgish writing system has some systemic peculiarities, for example, there is a contextual (phonetic) rule according to which the endings *-n* or *-nn* are not to be written before initial vowels and some consonants in the following word, the so-called “n-rule” (Zenter für d'Lëtzebuerger Sprooch, 2019).

In the following, we analyze the RTL user comments as for language attitudes. We use the articles only as a supplementary data source for preprocessing (i.e., learning of an additional embedding model for orthographic normalization). In a follow-up study, it would be worthwhile to look for systematic connections between journalistic reporting and user discussions.

#### Preprocessing

In view of the extent of linguistic variation, we develop a special preprocessing workflow for the user comments. The goal is to reduce the amount of variant spellings for lemmas in the data in order to obtain a smaller and semantically consolidated vocabulary for the analysis. The workflow includes cleaning the texts from special characters and markup language, sorting out non-Luxembourgish contributions through language detection, tokenizing the data, and orthographic normalization. We implement all work steps in *Jupyter Notebooks* with *Python 3*.

##### Cleaning of the data

Due to the origin of the texts (online news portal) and the period of their creation (2008–2018), the texts first have to be cleaned of special characters, incorrect encodings, and markup language. In addition, since its foundation, the news platform has undergone several changes in the technical basis, which are reflected in the data in the form of different markup standards. As a consequence, data cleaning has to deal with the removal of html tags and other markup elements for online texts, the conversion of various text encoding standards into Unicode characters, and also the removal of special characters and hyper-text content (links and other embedded elements). In order to find a tailored solution for the many encoding errors in the data, we use a dictionary-based approach to replace these characters.

##### Language detection

In a second step, we process all comments with the help of the package *langdetect* to identify the text language. For this purpose, we train a language profile for the recognition of Luxembourgish on the basis of the RTL news articles and implement it into the package. In this way, we can separate the Luxembourgish texts from comments in other languages. However, the recognition only works reliably on the comment level^1^. This preprocessing step reduces the amount of comments for the analysis to 544,143 posts. It also reduces the influence of multilingualism in the data. However, better language models are needed to process phenomena such as code-switching and loan vocabulary on the sentence level. For the further steps, this means that a certain number of foreign language elements remain in the text corpus (most of these words are filtered out by the frequency threshold during the training of word the word embedding model, though).

##### Tokenization

We then tokenize the data using the package *spaCy*. Since November 2019, this package has language support for Luxembourgish, including tokenization and POS tagging^2^. Compared to other resources (Sirajzade and Schommer, 2019), processing in *spaCy* works reliably for tasks like POS tagging, makes use of state-of-the-art algorithms and data formats, and also takes peculiarities of Luxembourgish spelling into consideration, such as the correct separation of *d'* as a definite article in words like *d'Saach* “the thing, the matter.”

##### Orthographic normalization

The most challenging step in data preparation is the orthographic normalization of the data. In view of the diverse sources of linguistic variation, we introduce the *Python* package *spellux*^3^, a pipeline that helps reducing the number of spelling variants in the corpus without having to exclude them for the subsequent training of a word embedding model (i.e., by setting a frequency threshold parameter). For this purpose, we use a multi-stage process, which compares a variant with different correction instances and, in unambiguous cases, corrects the text. Different correction resources are available for this task:

- *Word embedding model*: Based on the entire corpus, that is, user comments and news articles, we train a vector space model using the *gensim* package (word embedding with *Word2Vec*; Mikolov et al., 2013). The goal is to use representation learning to identify orthographically similar forms of the same lemma with the model. This is possible because word embedding models structure corpora in a high-dimensional vector space according to the *contextual similarity* of words based on semantic-syntactic co-currencies. The use of all data for the embedding model makes it possible to compare the individual spelling variants in the comments with the correct spellings in the articles—because they appear in comparable contexts in terms of linguistic structure. We use the following common hyperparameters to train the model (Mikolov et al., 2013; Pierrejean and Tanguy, 2018): dimensions: 200, window size: 5, iterations: 5, word frequency threshold: 25, downsampling of frequent words: 1e−3.
- *Correction dictionary*: We implement a list of lemmas and spelling variants from the online correction tool “spellchecker.lu.” With the help of this tool, writers can check Luxembourgish texts online and replace spelling mistakes with correct variants. The entered variants and correction lemmas are logged in the tool. We create a correction dictionary from these, which contains the most frequent (f > 20) spelling variants for each lemma as well as the summary correction frequency for all variants of a lemma (Note that this dictionary is only used for training the correction models in *spellux* and not part of the official release).
- *tf-idf matrix*: We train a tf-idf correction matrix using the entire lemma list from the correction dictionary as a basis, and the *TfidfVectorizer* method in the package *scikit-learn*. In doing so, we determine the k-nearest neighbor for a given variant in the lemma list.
- *Norvig spelling corrector*: Additionally, we implement an adaptation of the spelling corrector by Peter Norvig that has been tailored to Luxembourgish orthography^4^. The corrector evaluates the most likely correction candidate for a given variant based on a large text sample (of RTL news articles).

For orthographic normalization, we use the following workflow:

- First, we compare each word form with the lemma list in the correction dictionary. We classify variants recorded as lemma as correct (including some false positives for homographic forms).
- Second, we check whether forms that are not included in the lemma list are listed as spelling variants in the correction dictionary. If the form is recorded as a variant of exactly one lemma, we replace it with the corresponding lemma in the text. In cases where a form is used as a spelling variant for several lemmas (e.g., *as* for *ass* “is” and *als* “as”), we run an extended correction routine. To do so, we can choose from different correction modes (see the package documentation for further details): We can either check a variant for its vector similarity (*cos* θ) with all words in the word embedding model to determine a correction candidate by its contextual similarity with the variant, we can determine a candidate using the tf-idf matrix, we can evaluate a candidate using the Norvig corrector, or can we use a combination mode that accesses all three correction modes. To assure correction accuracy, and for best candidate evaluation in the combination mode, we evaluate the string similarity of correction candidates against the input form using the *Jaro Winkler* distance measure in the package *jellyfish*. In the event of a good enough match, we replace the variant with the best candidate. Given that the word embedding model was trained on the entire RTL corpus, we choose the embedding model as the default correction mode.
- If we cannot determine a clear candidate using the correction routine, the spelling variant is not corrected.
- We write each pair of spelling variant and lemma found to a dynamic matching dictionary to save the matches for later occurrences of the same variant and speed up text correction.

The comment corpus comprises 38,568,920 words. Through the orthographic normalization and case conversion, we reduce the number of unique words in the corpus from 1,102,377 to 1,017,175. Nevertheless, there are 680,300 unique words in the corpus for which we find no replacement using the available correction resources. Some of these are misspellings that are not yet recorded in the correction dictionary, some are words that are missing from the lemma list, some stem from foreign language material left in the comments (code-switching, citations). Further processing would be necessary for these words to improve the automatic normalization of the texts, for example, the semi-automatic extension of the correction dictionary by these variants.

#### Modeling

On the basis of the orthographically normalized texts, we train a new word embedding model (using the same training hyperparameters as before) that includes only the user comments. This model serves as the basis for the reconstruction of language attitudes toward multilingualism. According to the logic behind representation learning, the vectors of words that have a closer semantic-syntactic connection should have a higher contextual similarity in the vector space model. For example, in the data, the country name *Lëtzebuerg* “Luxembourg” is contextually more similar to the vector representation of its polity (*Monarchie* “monarchy,” cos θ = 0.260223) than to the vector for the word “democracy” (*Demokratie*, cos θ = 0.245135)—nevertheless, Luxembourg is of course a democratically governed country. However, we cannot interpret this relation as an exact representation of the semantic-syntactical closeness of the concepts in question. For example, the vector for *Diktatur* (“dictatorshop,” cos θ = 0.273865) is even closer to *Lëtzebuerg*.

Nevertheless, it is possible to interpret the contextual similarity of word vectors in the embedding model as statements about the relative *discursive proximity* of concepts in the dataset, for example, regarding language attitudes. Words whose vector representations are closer together in the model are more likely to appear in similar semantic and syntactic contexts—without necessarily specifying the exact quality of this relation. That is why we are interpreting this relation holistically, that is, as a combination of semantic (*concept similarity*) and syntactic (*context similarity*) information that, in sum, mirrors the sociopragmatic use of a word relative to others in the corpus. To avoid false conclusions, however, and given the general vulnerability of word embedding models to input variability and training hyperparameters, we compare the data with the results of a questionnaire survey on language attitudes. The comparison of the learned word representations and the empirically tested language attitudes makes it possible to draw conclusions about the representation and evaluation of languages in discourse, but also about the meaningfulness of the learned representations for the analysis.

#### Related Research

The general benefit of representation learning and distributional semantics for the reconstruction of the social meaning of concepts has already been examined in computational linguistics. Grondelaers and Speelmann (2015) use vector space models to cluster keywords returned in a free-response experiment on language attitudes into semantically meaningful dimensions for interpretation. Garg et al. (2018) demonstrate how the temporal encoding of word embedding helps to quantify changes in stereotypes and attitudes toward women and ethnic minorities. And Kozlowski et al. (2019) show that vector representations of semantic word relations in such models (e.g., for *man*—*woman, rich*—*poor*) can be related to common cultural stereotypes in public discourse. In addition, there are other approaches for determining attitudes and emotions in language data.

For example, Dong et al. (2019) show based on crowdsourced questionnaire data that the cross-cultural perception of social roles differs considerably and that these differences can be predicted using attributive descriptors or associated actions for social roles in context. Hassan et al. (2010) introduce a method to identify reciprocal attitudes of participants in an online discussion forum by evaluating positive or negative elements in sentences. The approach is expanded to the “AttitudeMiner” system in Abu-Jbara et al. (2012). Dasigi et al. (2012) automatically detect subgroups of users in online discussion threads based on implicit attitudes expressed by similar language use, similar to Somasundaran and Wiebe (2009) who focus on debate genre and opinion-based social stance in multiauthor threads. Rodríguez-Penagos et al. (2012) introduce a modular and scalable framework for opinion mining in social media data based on posts about Spanish telephone services and products. Lin et al. (2013) automatically track discussion dynamics in social media using topic-based attitude modeling and topical position mapping to determine the participants positionings toward each other. And Chuang and Hsieh (2015) perform a binary classification task to determine stances in social media posts with a lexicon-based approach that makes use of linguistic feature analysis and manual annotation.

There are also a number of earlier studies that employ different methods to try to determine the contextual emotional value of sentences in text data, be it with the help of keyword matching techniques (Chuang and Wu, 2004; Strapparava et al., 2007), calculations of emotion points (Taboada and Grieve, 2004), sets of linguistic interpretation rules (Boucouvalas, 2003; Chaumartin, 2007), sets of predefined attitude labels (Neviarouskaya et al., 2009), or machine learning methods (Aman and Szpakowicz, 2008; Strapparava and Mihalcea, 2008). Pang and Lee (2008) offer a comprehensive overview of early work on sentiment analysis and opinion mining.

So far there is hardly any comparable work for Luxembourgish, as well as for attitudes toward multilingualism in general. As part of the STRIPS project (Gierschek et al., 2019), we are currently developing an engine for automatic sentiment analysis for Luxembourgish. The system makes use of manually annotated training data, word embedding, and recursive neural networks for sentiment prediction.

What is striking about most computational linguistic work on the nexus *ideology*—*attitude*—*stance* –*sentiment*—*emotion* is the lack of a coherent conceptual basis that is grounded in linguistic and socio-psychological theory, and with it a clear delimitation of the different concepts involved (see for example Munezero et al., 2014). Often the terms for the examined concepts change several times within the same text. In this respect, the present study may also serve as a contribution to the theoretical foundations of computational sociolinguistics with regard to the social meaning of linguistic phenomena in interaction. In many studies, there is also a problematic equation of observable language use (i.e., stance, sentiment) and the assumed underlying cognitive entities (i.e., attitude, emotion), while the social-psychological literature on attitudes particularly emphasizes the lack of a direct attitude-action link. In addition, many studies seem to be primarily interested in the technical aspects of the implementation, prediction accuracy, and evaluation of methods for the detection of emotions or opinions in utterances, less in their applicability to and meaningfulness for sociolinguistic research. Against this backdrop, the combination of different data types for the purpose of a sociolinguistic analysis of attitudes is particularly worthwhile.

### Crowdsourcing Attitudes With the “Schnëssen” App

#### Dataset

The data for the sociolinguistic analysis stem from a questionnaire survey as part of the crowdsourcing project “Schnëssen” (Entringer et al., forthcoming). The project is an initiative of the Institute for Luxembourgish Language and Literature at the University of Luxembourg and aims to document variation and change in present-day spoken Luxembourgish. For this purpose, we have developed a mobile research app with which speakers of Luxembourgish can record their own language use. Since 2018, we have collected voice data from more than 2,500 speakers and for more than 500 linguistic phenomena in this way. In addition to the language survey, a sociolinguistic questionnaire can also be accessed via the app, which specifically asks about the participants' attitudes to multilingualism and Luxembourgish. We use a specially developed quantitative instrument to collect the attitudes.

Participants are asked to rate comments on five-tier Likert scales. In contrast to comparable studies, we take care to ensure that the statements to be assessed mirror situations that respondents are familiar with and encounter frequently in everyday life. A general weakness of quantitative attitude measurements should be avoided in this way (see Purschke, 2014 for a discussion): Comparable studies often ask about abstract concepts or assessments for which there is no direct correspondence in the everyday experience of the respondents. As a consequence, in many cases, the respondents must first form an opinion to the subject of the question instead of activating their existing everyday knowledge.

The questionnaire covers four thematic areas: the development of multilingualism in the country, the state of Luxembourgish, the social presence of the most important languages, and individual language preferences in everyday situations. Between April and January, 2019, 2,158 complete questionnaires have been collected that can be used for the analysis. In addition, each participant has created a social profile in the app that contains the most important biographic and linguistic information. This includes language skills, places of residence, stays abroad, educational profile, age, and gender. In view of the technical and linguistic requirements of the app, the data shows a characteristic demographic bias: The app is entirely in Luxembourgish and also requires knowledge of German and French for translation tasks. As a consequence, the app has linguistic preconditions that are primarily met by Luxembourgish native speakers, who make up more than 90% of the sample, whereas the other half of the population is hardly represented. In addition, there is the usual demographic bias for app-based surveys that rely on voluntary work, that is, young, well-educated, female participants are overrepresented in the sample (Behrend et al., 2011).

#### Preprocessing

In order to prepare the data for analysis, we have to match the questionnaire data with the users' social profiles (using a device-specific unique identifier). The reason for this lies in the fact that the questionnaire is embedded in the app as an independent task, but the creation of a social profile is only mandatory for the app's recording function. As a consequence, many participants filled out the questionnaire without creating a social profile. In addition, there are cases in which several people made recordings or filled out the questionnaire using the same device, which is why sometimes there are several social profiles and only one questionnaire for the same universal identifier and vice versa. To deal with this situation, we first match the unique questionnaires and unique social profiles. The remaining cases of doubt, in which the number of social profiles and questionnaires differ, we match manually if possible. After preprocessing, 1,832 completed questionnaires remain, which can be assigned to a unique social profile. These data form the basis for the following analysis.

#### Related Research

So far, there are only a few studies on attitudes and stances toward Luxembourg multilingualism. These focus primarily on the language preferences of speakers in various everyday situations, for example, in work contexts or leisure activities (Fehlen, 2009; Fehlen and Heinz, 2016). The studies show a clear connection between (first) language competence and language preference in practice. In addition, the practical requirements of everyday life play a central role in the situational choice of a language. Conrad (2017) includes similar questions in the analysis of contact-related variation in Luxembourgish to explain the preference of the speakers for Germanic or Romance variants in use. Redinger (2010) deals with language attitudes and language behavior in the Luxembourg educational system in combing a questionnaire survey with an ethnographic investigation of in-class code-switching. Wagner (2012, 2013) investigates writing strategies and their relation to language use and ideologies in social media discussions on Facebook. In a similar vein, Belling and de Bres (2014) investigate the role of Luxembourgish for group negotiations and identity constructions in a multilingual Facebook group. Language ideologies and the practical negotiation of multilingualism in the workplace, with particular attention to cross-border commuters, are the focus of the studies by Franziskus (2013), De Bres (2014), and De Bres and Franziskus (2019). Lately, Bellamy and Horner (2018) focus on ideological positionings in interaction with regard to the societal role and linguistic status of Luxembourgish as a national language. In a questionnaire survey with more than 2,000 participants, Stölben (2019) examines the Luxembourgers' attitudes toward the official languages in the country, with a special focus on German. The study documents the complex attitudinal horizon of the Luxembourgers regarding the different languages in the country, with both the domain-specific organization of multilingualism as well as individual factors such as language competence and social environment contributing to individual attitudes.

All studies establish a clear connection between language competence, language preference, and sociocultural orientation in everyday life. The role of Luxembourgish as a practical means of individual social positioning (*identity level*) and a symbolic resource of group-related identification (*ideology level*) is particularly important in this context. For the study of language attitudes, this means that the position of Luxembourgish in the complex Luxembourg multilingualism is crucial, but also the structure and dynamics of the language regime as a whole.

## Results

Based on these findings, we present selected results of the questionnaire survey below and contrast them with queries to the word embedding model trained on the user comments. Since the comments are free text data that represent reactions to journalistic content, many texts contain clear positive and negative stances on certain topics that seem suitable for the aggregating reconstruction of attitudes. *Example 2* gives another example of such public stances in the dataset that also illustrates the difference between *explicit* and *implicit* aspects of stances and attitudes in practice: first, the author explicitly positions themselves in favor of Luxembourgish by calling for resistance (*Fannen och mir mussen ons wiehren*. “I also think we must fight back.”), followed by a direct call for action (*Rett ons sproch* “Save our language”. Then, in addressing the audience they code-switch from Luxembourgish to English (*be united people*). In view of the language-ideological subject of the comment, the switch to English is likely to take place at an implicit level of stance-taking, also because code-switching is part of the highly routinized repertoire of multilingual speakers in Luxembourg.

**Example 2: Language-related comment from the RTL data set**
*Fannen och mir mussen ons wiehren. Rett ons sproch, be united people* [2016-02-21]
**Translation:** I also think we must fight back. Save our language, be united people

The results of the computational text analysis are not to be equated with the quantitatively surveyed attitudes in the questionnaire, though. By comparing the two datasets, however, we can draw conclusions concerning attitudes toward multilingualism present in the Luxembourg population. Comments and survey data serve as complementary data sources that link publicly taken stances in discourse to underlying attitudes that impact the structure and dynamics of the language regime in the country. For example, the growing discussion about the societal role of Luxembourgish in recent years has had a direct impact on politics, which was reflected in the issue of language as a topic in the national election campaign in 2018 as well as in the newly introduced language promotion law for Luxembourgish. Connecting these two datasets is the particular challenge—and the particular contribution—of the following computational sociolinguistic analysis.

### The Social Presence of Languages in the Language Regime

The first set of results relates to the social presence of the various languages in the country, that is, their position and symbolic value in the language regime. There are a couple of questions in the questionnaire that are of interest in this context. This includes the question of which of the most important languages “belong” to the country (Table 1). So, the question is about the cultural self-image of the Luxembourgers with respect to languages. The results show that Luxembourgish is widely identified as the language that belongs the most to Luxembourg. There is also a majority which identifies French and German, the other two official languages, as belonging to the country as well. In contrast, Portuguese, the strongest minority language in the country (16% of the total population have Portuguese roots; STATEC, 2019), is not largely attributed to the country. Compared to English, however, for which the answers show a symmetrical distribution (which indicates indecision among the respondents), it belongs more to Luxembourg.

**Table 1:** Belonging of the most important languages to Luxembourg | *N* = 1,831, *p* < 0.001 (χ^2^).

**“… belongs to Luxembourg”**	**Agree (%)**	**Somewhat yes (%)**	**Neither nor (%)**	**Somewhat no (%)**	**Disagree (%)**
Luxembourgish	91.1	6.5	1.1	1.0	0.2
French	36.6	41.9	8.8	6.2	6.4
German	25.6	47.2	13.2	9.9	4.1
English	9.3	30.0	22.6	27.7	10.5
Portuguese	13.7	35.3	17.2	17.3	16.5

We also find this clear hierarchy of languages present in the country in the aggregated user comments from RTL, as a query of the vector similarities to the country name *Lëtzebuerg* for the same five languages shows:

*Lëtzebuergesch* (“Luxembourgish”, 0.368894), *Franséisch* (“French”, 0.296720), *Däitsch* (“German”, 0.288161), *Englesch* (“English”, 0.276643), *Portugisesch* (“Portuguese”, 0.272050)

Remember that the closer a word vector for a language in the model is to the comparison vector, the higher its discursive proximity, that is, its likelihood of appearing in comparable semantic-syntactic contexts, for example, discussions about multilingualism. The query results show that the three-tier hierarchy of languages established in the survey data is also present in the aggregated user comments, with *Lëtzebuergesch* being the closest to *Lëtzebuerg*, followed by *Franséisch* and *Däitsch*, and *Englesch* and *Portugisesch* at a greater distance.

This connection becomes even clearer when asking about the presence of the different languages in everyday life, for example in the public. Traditionally, the majority of public writing is in French and German, but in recent years there has been a substantial increase in Luxembourgish (due to its societal revaluation) and English (as a sign of internationalization).

This aspect of discourse is reflected in the embedding model, for example, in the vector similarities of the variants *Public* (“the public” Romance origin), Ë*ffentlechkeet* (“the public” Germanic origin), and Alldag (“everyday life”) for the same languages:

- **Alldag**: *Englesch* (0.274927), *Franséisch*, 0.241679), *Lëtzebuergesch* (0.233781), *Däitsch* (0.191266), *Portugisesch* (0.089524)
- **Public**: *Lëtzebuergesch* (0.114520), *Franséisch* (0.081171), *Englesch* (0.048003), *Portugisesch* (0.032752), *Däitsch* (−0.030943)
- **Ëffentlechkeet**: *Englesch* (0.158184), *Lëtzebuergesch* (0.152099), *Franséisch* (0.111701), *Däitsch* (0.044319), *Portugisesch* (−0.0518915)

In all cases, German and Portuguese occupy the lower places, which above all reflects the fact that both languages are hardly discussed in the discourse. In contrast, Luxembourgish and English (on the upswing), together with French (perceived as too strongly present), form the discursive center of the discussion about the languages in the country. If we query specific aspects of written language in public, on the other hand, for example for *Stroosseschëlder* (“street signs”), we get an accurate ranking of the presence of the different languages in the public sphere (see Purschke, 2020 for a quantitative survey of the Luxembourg City linguistic landscape):

- **Stroosseschëlder:** *Franséisch* (0.255751), *Däitsch*, (0.240960), *Lëtzebuergesch* (0.240203), *Englesch* (0.205646), *Portugisesch* (0.187109)

There is a societal demand for a greater presence of Luxembourgish in the public sphere, which is also related to the demographic development of the country, and which is reflected in the survey data in the question of which languages should be more visible in public (Table 2). The vast majority of respondents expresses a wish for Luxembourgish to have a greater presence as opposed to the other languages in question. The respondents in particular reject French, which has been assigned a problematic role in the public discussion due to its strong presence among the foreign working population, and Portuguese, which is identified as a language linked to migration in Luxembourg.

**Table 2:** Language visibility in public space | *N* = 1,831, *p* < 0.001 (χ^2^).

**“… should be more visible in public space”**	**Agree (%)**	**Somewhat yes (%)**	**Neither nor (%)**	**Somewhat no (%)**	**Disagree (%)**
Luxembourgish	76.6	16.3	6.2	0.4	0.5
French	1.9	7.2	36.2	28.6	26.0
German	5.8	15.2	40.0	23.0	15.9
English	7.0	18.4	35.2	21.3	18.1
Portuguese	0.6	3.8	22.9	24.1	48.5

### The State of Multilingualism

Another section of the questionnaire deals with the assessment of the situation of multilingualism in the country. In this context, we asked the respondents a three-part question that addresses different attitude-related aspects. First the participants had to assess the *current state* of multilingualism. Second, the participants should assess a prognostic statement regarding the future development of multilingualism (*future state*). And third, we used a statement on the preservation of multilingualism in the country to establish the normative dimension (*target state*) of attitudes. By comparing the different answers, we can determine the *attitudinal horizon* of the respondents regarding this complex (Table 3).

**Table 3:** The state of multilingualism | *N* = 1,825, *p* < 0.001 (χ^2^).

**“Multilingualism in Luxembourg…”**	**Agree (%)**	**Somewhat yes (%)**	**Neither nor (%)**	**Somewhat no (%)**	**Disagree (%)**
Is functioning without problems	16.7	47.6	13.6	16.1	6.0
Will function without problems in the future	15.3	42.1	15.0	20.9	6.7
Should remain	50.1	33.2	7.7	6.0	3.1

The results show that Luxembourgers in general have a positive attitude toward multilingualism. A large majority of respondents want it to persist. A majority of the participants also make a positive assessment of the current situation and future development of multilingualism. However, this result also shows that, on the one hand, a substantially larger proportion of the respondents (~25% each) also see problems in this context, and, on the other hand, the respondents assess the future development of the situation slightly more skeptically than the current state (we make the same observation for similar questions in the study).

A potential reason for the shape of this attitudinal horizon can be found in the comment data. The analysis of the 10 nearest word vectors to the term *Méisproochegkeet* “multilingualism” points to several discursive contexts:

*Villsproochegkeet* (“multilingualism”, 0.738968), *Identitéit* (“identity”, 0.654430), *Nationalsprooch* (“*national language*”, 0.645417), *Bankeplaz* (“*banking center*”, 0.629268), *Souveränitéit* (“*sovereignity*”, 0.627935), *Sprooch* (“*language*”, 0.623087), *Orthographie* (“*orthography*”, 0.618942), *Ekonomie* (“*economy*”, 0.609412), *Zivilisatioun* (“*civilization*”, 0.609396), É*conomie* (“*economy*” Romance variant, 0.605071)

First, we see a close relationship with other language-related concepts, which can be expected due to the model logic of word embedding. Second, and more interestingly, multilingualism appears in a discursive context that deals with societal and national issues (*Identitéit, Souveränitéit*). Against the backdrop of the public discourse on the language situation in recent years, this shows above all the close connection between language- and identity-related questions that partly shape the public discussion in Luxembourg, especially in political and right-wing populist contexts. Third, the word vectors that refer to economic aspects (*Ekonomie, Bankeplaz*) demonstrate the close interdependence of the Luxembourg economic model with multilingualism: the private sector and the financial industry mostly employ foreign workers. The increase in this population group through migration and cross-border commuting, as well as the associated presence of languages other than Luxembourgish in public, are the rated breaking points in the societal discussion on multilingualism.

### The Status of Luxembourgish

Another central issue in the public discussion concerns the role of Luxembourgish, that is, its status as a language. Linguistically speaking, Luxembourgish is a Moselle-Franconian dialect and is therefore closely related to the German regional languages (Gilles, 2019). Despite the fact that Luxembourgish has been declared the national language by law in 1984—and thus has an official language status—there are still stances in the discourse that describe Luxembourgish as a German dialect (as opposed to German and French as fully-developed and prestigious languages of culture; Sieburg and Weimann, 2014). However, if we ask the participants about the status of Luxembourgish, a large majority confirm its official status as a language (Table 4). At the same time, 20% of the respondents only somewhat agree to the question. This assessment coincides with further judgments about the status of Luxembourgish in the data: an equally large majority supports the recognition of the language at EU level. In addition, there is a clear positioning (and expectation of linguistic integration) vis-à-vis immigrants with regard to language acquisition (remember the stance in *Example 1*).

**Table 4:** The status of Luxembourgish *N* = 1,829, *p* < 0.001 (χ^2^).

	**Agree (%)**	**Somewhat yes (%)**	**Neither nor (%)**	**Somewhat no (%)**	**Disagree (%)**
“Luxembourgish is an independent language”	73.9	20.0	3.6	2.1	0.4
“Luxembourgish should be officially recognized as language of the EU”	69.6	15.6	6.2	4.9	3.6
“Newcomers to Luxembourg should learn Luxembourgish”	61.2	31.3	6.6	0.6	0.3

Contrasting the respondents' attitudinal horizon regarding Luxembourgish with the public stances in the comment data also reveals a correspondence. In the aggregated data there is a greater discursive proximity from *Lëtzebuergesch* to the vector for *Sprooch* (“language,” 0.642606) than to the vector for *Dialekt* (“dialect,” 0.487487). This means that Luxembourgish is discussed more likely in the context of a (national) language than its origin as a dialect of German.

A characteristic (and strength) of Luxembourgish is its high degree of linguistic plasticity. The language has a high proportion of elements of German or French origin and continues to integrate them without problems. In the current discourse climate, however, this flexibility is sometimes seen as problematic, for example by language activists who are committed to keeping Luxembourgish “clean” from “foreign” influences. A good indicator question in the questionnaire for this connection is that of the assumed linguistic influences on Luxembourgish in the future (Table 5). As can be seen, the respondents see a growing influence of English and French on the language, not so much of German. Interestingly, this assessment somewhat contradicts linguistic reality. As Conrad (2017) shows, younger speakers in particular show a clear preference for the Germanic variants when choosing between parallel phonological variants, not toward the Romance variants. In this respect, we can read the result as an assessment of the *assumed cultural influence* of the languages in Luxembourg society rather than of their *factual linguistic influence* on Luxembourgish.

**Table 5:** Future influences on Luxembourgish | *N* = 1,826, *p* < 0.001 (χ^2^).

**“The influence of … on Luxembourgish will grow in the future”**	**Agree (%)**	**Somewhat yes (%)**	**Neither nor (%)**	**Somewhat no (%)**	**Disagree (%)**
German	4.0	22.7	34.3	31.3	7.7
English	8.2	42.2	20.8	20.1	8.7
French	11.0	41.1	26.8	16.2	4.9

Again, we can see the same assessment in the comment data. Querying for the 20 nearest neighboring vectors for different combinations of *Lëtzeguergesch* + x (i.e., *Afloss* “influence,” *Entwécklung* “development,” *Zukunft* “future”), it becomes apparent that French and English are always in a greater discursive proximity than German:

- **Afloss**: ***Franséisch*** (“French”), ***Englesch*** (“English”), ***Franzéisch*** (“French,” spelling variant), *Impakt* (“impact”), *Zougrëff* (“access”), **franséisch** (“French,” ADJ/lower case N), ***Franséich*** (“French,” spelling variant), *Accent* (“accent,” Romance variant), *Akzent* (“accent,” Germanic variant), ***englesch*** (“English,” ADJ/lower case N), ***D*ä*itsch*** (“German”), *Letzebuergech* (“Luxembourgish,” spelling variant), *Lëtzbuergesch* (“Luxembourgish,” spelling variant), Lëtzebuergescht (“Luxembourgish,” inflection form), *Urecht* (“entitlement to”)
- **Entwécklung**: *Sprooch* (“language”), *Orthographie* (“orthography”), *Integratioun* (“integration”), ***Franséisch*** (“French”), *Schreifweis* (“spelling”), ***Englesch*** (“English”), *Grammatik* (“grammar”), *Allgemengbildung* (“general education”), *Literatur* (“literature”), *Rechtschreiwung* (“orthography”), ***Franséischt*** (“French,” inflection form), *Lëtzebuergescht* (“Luxembourgish,” inflection form), *Kommunikatioun* (“communication”), *Evolutioun* (“evolution”), *Mammesprooch* (“mother tongue”)
- **Zukunft**: ***Franséisch*** (“French”), *Sprooch* (“language”), ***Englesch*** (“English”), *Mammesprooch* (“mother tongue), ***Sprooche*** (“languages,” n-rule form), *franséisch* (“French,” ADJ/lower case N), ***L*ë*tzebuergescht*** (“Luxembourgish,” inflection form), *sprooch* (“talk,” imperative/“language,” lower case N), ***englesch*** (“English,” ADJ/lower case N), *Integratioun* (“integration”), *Landessprooch* (“national language”), ***Franzéisch*** (“French,” spelling variant), ***Franséich*** (“French,” spelling variant), *Sproochen* (“languages”), ***Franséischt*** (“French,” inflection form)

Apart from the fact that in a word embedding model the different language names are inevitably close to each other (due to concept similarity), the different sequences and constellations indicate similar prognostic evaluations regarding the development of Luxembourgish. Ultimately, these constellations in the discourse mirror assumptions about the *global cultural dynamics* of the country (demographically and economically), and the languages are representative of this.

### The Language-Identity Link

The comment data in particular reveal a close connection between linguistic concepts and those that belong more in the area of identity and nationality. For the 30 closest neighbors to the word vector *Sprooch* “language,” the embedding model shows elements that we can link to different semantic domains (Table 6).

**Table 6:** Semantic domains of nearest neighbors to *sprooch* “language.”

**Linguistic concepts**	***Sprooch* (“talk,” imperative/“language,” lower case N), *Schreifweis* (“spelling”), *Sproch* (“language,” spelling variant, “saying”), *Orthographie* (“orthography”), *Friemsprooch* (“foreign language”), *Sproochen* (“languages”), *Sprooche* (“languages,” n-rule form), *Grammatik* (“grammar,” Germanic variant), *Grammaire* (“grammar,” Romance variant), *Méisproochegkeet* (“multilingualism”), *Rechtschreiwung* (“orthography”), *Villsproochegkeet* (“multilingualism”), *Weltsprooch* (“world language”), *Ëmgangssprooch* (“colloquial language”), *Mondart* (“dialect”), *Iwwersetzung* (“translation”)**
Language concepts	*Lëtzebuergesch* (“Luxembourgish”), *Franséisch* (“French”), *Lëtzebuergescht”* (“Luxembourgish,” inflection form)
National concepts	*Landessprooch* (“national language”), *Nationalsprooch* (“national language”), *Nationalitéit* (“nationality”), *Amtssprooch* (“official language”), *Gesetzgebung* (“legislation”), *Nationalhymne* (“national anthem”), *Verfassung* (“constitution”), *Integratioun* (“integration”)
Identity concepts	*Mammesprooch* (“mother tongue”), *Identitéit* (“identity”), *Kultur* (“culture”)

In addition to the language names for French and Luxembourgish (not German, though!), there are a number of other related concepts that we can assign to the linguistic context of the term *Sprooch*, including *Grammaire/Grammatik* “grammar,” W*eltsprooch* “world language,” or spelling variants and inflection forms of the concept. However, there are also a number of concepts that place the word in other semantic domains, namely references to words that relate to political and nation-state contexts, and words that relate to individual or collective identity constructions. This discursive proximity of different semantic domains also indicates the range of possible discursive contexts in which the concept of language appears in the comment data. In this context, we can read the identity- and nation-related concepts as an indication of the close connection of language, identity, and nation in the discourse, which is in fact a characteristic of the public discussion about language in recent years. Garcia (2014) diagnoses a strong politicization and ideological charging of the language discussion in Luxembourg. In this context, it is also revealing to observe that many Luxembourgers, when referencing Luxembourgish, use the term *eis Sprooch* “our language” (see above, *Example 2*), that is, they directly identify the language with the political community—as opposed to the other official languages of the country, French and German.

We find an additional illustration of this nexus by querying the vector similarities for the concepts *Mammesprooch* “mother tongue” and *Friemsprooch* “foreign language” with the vectors for the most important languages:

- ***Friemsprooch***: *Franséisch* (0.562637), *Englesch* (0.552961), *Portugisesch* (0.540379), *Däitsch* (0.516047), *Lëtzebuergesch* (0.507465)
- ***Mammesprooch***: *Lëtzebuergesch* (0.553955), *Franséisch* (0.547082), *Däitsch* (0.517634), *Portugisesch* (0.512621), *Englesch* (0.510809)

As we can see, the contextual similarity is different for the two concepts, with Luxembourgish being closest to the concept mother tongue and furthest away from the concept foreign language, unlike English. German and Portuguese occupy middle positions in both queries. A possible reason for this could again be the fact that these languages are not assigned a problematic role for the organization of multilingualism in the current discourse. Most interestingly, French is close to both of the concepts queried, reflecting its overall prominent role in the discourse: the language is seen as both “foreign” (linked to work-related migration) and “native” (historically rooted in Luxembourg multilingualism).

### Language Preferences in Everyday Practice

The close connection between language and self-image is not only evident in the discussions about language, but also in everyday preferences for certain languages. We asked a number of questions in the questionnaire that not only provide information about specific language preferences, but also demonstrate that the language regime in Luxembourg is currently on the move. For example, the participants were asked which languages are important to them in everyday life (Table 7).

**Table 7:** General language preference in everyday life | *N* = 1, 824, *p* < 0.001(χ^2^).

**“… is an important tool for me in everyday life”**	**Agree (%)**	**Somewhat yes (%)**	**Neither nor (%)**	**Somewhat no (%)**	**Disagree (%)**
Luxembourgish	87.0	10.7	1.0	1.0	0.3
French	35.1	42.9	9.0	6.8	6.2
German	18.5	31.9	18.1	23.5	8.0
English	13.7	24.0	19.7	27.9	14.8
Portuguese	1.5	5.1	7.5	21.2	64.7

As the data show, there is a clear hierarchization of the different languages in terms of their practical use in everyday life, with Luxembourgish being by far the most important tool in practice. This statement also partially reflects the composition of the sample: the majority of the study participants are native Luxembourgers with Luxembourgish as (one of) their mother tongue(s). In addition, the data also confirm the important role of French in Luxembourg multilingualism. More interesting than the general usefulness are therefore the questions about the specific language preferences in everyday situations, for example, when watching TV news (Table 8).

**Table 8:** Language preference when watching TV news | *N* = 1,827, *p* < 0.001 (χ^2^).

**“Which language do you prefer when watching the news on TV?”**	**Luxembourgish (%)**	**German (%)**	**French (%)**	**English (%)**	**Portuguese (%)**	**Italian (%)**
1st choice	68.3	23.4	4.6	3.3	0.4	0.0
2nd choice	17.9	61.7	11.6	7.7	0.8	0.2
3rd choice	8.6	11.2	46.8	31.7	1.0	0.6

On the one hand, it becomes clear that the respondents do in fact have a strong preference for Luxembourgish (1st choice), but there is also an effect of the domain specificity of Luxembourgish multilingualism: In practice, many Luxembourgers mainly watch German television (2nd choice), partly because of the linguistic proximity to Luxembourgish, but also because the number of Luxembourgish channels is limited (to RTL). On the other hand, the 3rd choice is particularly interesting, in which the test subjects mostly choose between English and French. While the summary result seems to prefer French as 3rd choice, a look at the answers of the different age groups (Table 9) shows that the preference shifts from French to English with decreasing age.

**Table 9:** Language preference TV news, 3rd choice by AGE | *N* = 1,827, *p* < 0.001 (χ^2^).

**“Which language do you prefer when watching the news on TV?”**	**Luxembourgish (%)**	**German (%)**	**French (%)**	**English (%)**	**Portuguese (%)**	**Italian (%)**
≤24	9.3	10.0	36.1	41.1	2.4	0.7
25–34	5.5	11.2	45.4	36.6	1.1	0.2
35–44	8.3	8.9	50.5	31.5	0.6	0.3
45–54	9.3	13.4	56.0	19.4	0.0	1.4
55–64	10.3	11.8	54.9	21.5	0.5	1.3
≥65	20.5	17.9	47.4	14.1	0.0	0.0

We can compare these preferences with the RTL authors' language choices in the comment data, since writing a comment online also represents a (media-related) everyday situation. However, since writing in Luxembourgish is still a challenge for many Luxembourgers, this situation is far less routinized than watching TV news. On the other hand, the choice of language is influenced in part by the larger communicative context of the platform with Luxembourgish as default language for both news texts and comments. Based on the automatic language detection and considering only texts with more than 200 characters (see *Footnote 1* for information on detection accuracy), we find that the vast majority of texts is written in Luxembourgish (343,336 of 357,163 texts total), as opposed to 10,268 texts in German, 2,915 in French, and 399 in English—the remaining texts are mostly wrongly identified Luxembourgish texts labeled as Dutch. This result proves that—at least on the RTL platform—Luxembourgish has established itself as the default written language, but it also shows that German is preferred over French as an alternative language.

More generally speaking, and in line with most processes of language change, the age of the speakers is a determining factor for their linguistic orientation in everyday life—and thus for attitudes toward Luxembourg multilingualism. In the questionnaire data, age is the main demographic structuring factor explaining differences in attitudes. We can assume that the language regime will shift substantially in favor of English in the next few years, especially through the shift in the linguistic preferences of the young speakers—but also in view of the continuing internationalization of the resident population. In 2019, there was even a public petition to establish English as an official language in administrative contexts next to French and German^5^. In view of the many languages and sociocultural factors involved in this dynamic, it is hardly possible, though, to make a forecast about the development of the language regime as a whole.

## Discussion

Following the analysis, we discuss some methodological aspects in more detail below. This concerns the reconstruction of attitudes with the help of word embedding models as well as the collection of language attitudes data using crowdsourcing, but also the automatic orthographic normalization of Luxembourgish texts and potential limitations of the overall approach.

### Reconstructing Attitudes Using Representation Learning

The comparative analysis of attitudes toward multilingualism in Luxemburg has shown that word embedding models can be successfully used for the reconstruction of attitudes in free text data. The quantitative modeling brings to light discursive attitudinal patterns that represent the sum of many individual stances, without each individual stance itself necessarily being a direct expression of the aggregated attitude. During the preprocessing of the data, however, we have seen that and to what extent word embedding models are susceptible to the selection of the hyperparameters for training, that is, the number of vector dimensions or the window length for word contextualization (Goldberg, 2017; Pierrejean and Tanguy, 2018). The same holds true for data-intrinsic factors like the total number of words, vocabulary size, and word frequency range. Depending on the setting of the hyperparameters, different training results can be expected, especially in the upper and lower frequency range of the vocabulary.

In this respect, the orthographic normalization of the texts before training the data has a clear impact on the word embedding model on which the analysis is based. However, the comparison of different model solutions shows that the vector space is relatively stable for the concepts discussed in the present study, since it is usually a matter of words in the middle range of the frequency spectrum. For example, the 10 nearest-neighbor vectors for the word *Sprooch* “language” largely match before and after the orthographic correction:

- **Before normalization:** *Sproch* (“saying, language,” spelling variant, 0.842746), *Mammesprooch* (“mother tongue,” 0.800106), *Landessprooch* (“national language,” 0.769282), *Schreifweis* (“spelling,” 0.711668), *Nationalsprooch* (“national language,” 0.709543), *Identitéit* (“identity,” 0.700674), *Mammesproch* (“mother tongue,” spelling variant, 0.696856), *Orthographie* (“orthography,” 0.691917), *Mammensprooch* (“mother tongue,” spelling variant, 0.681196), *Sproochen* (“languages,” 0.673093)
- **After normalization:** *Mammesprooch* (“mother tongue,” 0.814756), *sprooch* (“talk,” imperative/“language,” lower case N, 0.771097), *Landessprooch* (“national language,” 0.759516), *Schreifweis* (“spelling,” 0.751803), *Sproch* (“saying, language,” spelling variant, 0.723642), *Nationalsprooch* (“national language,” 0.723429), *Orthographie* (“orthography,” 0.701390), *Identitéit* (“identity,” 0.692551), *Friemsprooch* (“foreign language,” 0.660245), *Nationalitéit* (“nationality,” 0.656720)

While the nearest neighbors represent more or less the same concepts, the example also demonstrates the value of orthographic normalization. After the correction process, several spelling variants are no longer among the nearest neighbors (and no longer in the vocabulary of the model). Nevertheless, orthographic normalization brings with it some methodological and practical challenges, for example, the lack of distinction between *Sproch* as a common spelling variant of *Sprooch* “language” and as a separate lemma with the meaning “saying.”

### Orthographic Normalization

Given the diverse sources of orthographic variation in Luxembourgish, the normalization of the texts is an important step in preparing the data for analysis. Normalization (using the current build of the *spellux* package) reduces the number of unique words in the data set and ensures more consistent vector representations by integrating orthographic variants into the basic lemma. The pipeline developed for processing the data works reliably, but the correction does not produce error-free texts. On the one hand, this is due to the number of orthographic variants that are not yet captured by the correction resources. On the other hand, the correction routine also produces a number of *false positives* and *false negatives*: Some words that can be identified in context as misspellings of lemmas also exist as an independent lemma with a different meaning (remember the example *Sprooch*—*Sproch*). In this case, we do not correct the word, due to a false-positive validation of the word form in the lemma list. At the same time, in the course of normalization, we do correct a number of word forms that represent misspellings to lemmas that are either contextually incorrect (because the word form is listed as a variant in the correction dictionary) or wrongly evaluated as a correction candidate in the comparison with the correction resources. As for the peculiarities of the writing system (n-rule), the *spellux* package has a dedicated rule-based correction routine for this context rule. Given the large amount of exceptions from the base rule (e.g., for personal and country names), however, we still cannot capture all cases when automatically correcting texts. We must therefore establish criteria for orthographic normalization to evaluate the advantages and disadvantages of an automated text correction, also in light of its impact on model training.

The comparison of the corrections made to an example text is helpful for illustration of the effects and challenges of automatic normalization. Misspellings in the original text are marked in *italics* (including n-rule errors). Correct corrections in the normalized text are marked in **bold**, incorrect corrections are underlined and variants that have not been corrected remain in *italics*.

**Before Normalization:**
*Den* Grand-Duc huet gerad *eso* Recht fir no sengem *Gewessen* ze entscheeden, *an* wann dat *den sogenannte* Spëtzepolitiker *an verschiedene* Journalisten net *gefaellt* dann *haet schons laengst* versicht solle *gin* Verfassung *dementsprechend* ze *aenneren*. *Den* Problem do, huet eng *Kéer missen kommen.An dann welle* Politiker an *eso* engem Dossier *wei* Liewen an den Doud *Haptwuert huen*, mat engem *débat doriwer wo* sie den Niveau *emmer mee* erof *zéen an* Leit um *terrain kennen* herno kucken dass se kloer kommen
**After Normalization:**
**De** Grand-Duc huet grad **esou** Recht fir no sengem **Gewëssen** ze entscheeden, **a** wann dat **de sougenannte** Spëtzepolitiker **a verschidde** Journalisten net **gefält** dann **hätt schonns längst** versicht solle **gi** Verfassung **deementspriechend** ze **änneren**. **De** Problem do, huet eng *Kéer* **misse kommen. An da wëlle** Politiker an **esou** engem Dossier *wei* Liewen an den Doud *Haptwuert* **hunn**, mat engem *débat* **doriwwer** *wo* Sie den Niveau **ëmmer** *mee* erof **zéien a** Leit um **Terrain** *kennen* herno kucken dass se kloer kommen.

As we can see, the automatic correction replaces most of the incorrect spellings with the correct ones. In addition, there are also some false corrections, e.g., *sie*_[before]_ (“they”) is corrected to *Sie*_[after]_ (“B,” musical note, plural + n-rule reduction) instead of the correct pronoun spelling *si*. No correction was made to some variants, be it because no variant–lemma pair was found in the correction resources (*Kéer* for *Kéier* “time, occasion”), be it because the variant matches with the wrong lemma in the lemma list (*mee*, meaning *méi* “more” in this context, matches with the lemma *mee* “but”). For these cases, we must expand the correction dictionary with additional spelling variants and finetune it. A final type of change relates to the form *kommen.an* in the original text. This is an artifact of tokenization and is detected during the correction routine. Regardless of such problems, the current correction architecture can already substantially consolidate the vocabulary of the data set.

A number of factors must be taken into account for further developing the *spellux* package:

- We must expand the correction dictionary to include more spelling variants that are present in the data but have not been recorded so far to reduce the number of unidentifiable variants.
- We must evaluate the use of case-sensitive models for correction and training: while the current workflow increases the number of remaining spelling variants in the corpus (e.g., *Lëtzebuergesch* N vs. *lëtzebuergesch* ADJ/lower-case N), using a lower-case model would produce a higher number of homographic lemmas and therefore reduce correction accuracy.
- We should integrate additional contextual cues to word disambiguation in order to determine correction candidates for variants without corresponding lemma in the existing correction resources. This includes candidate evaluation based on POS tags as well as on n-grams.
- We should systematically evaluate the training parameters for the correction resources with regard to their impact on correction performance. This applies above all to the correction frequency threshold for the spelling variants when building the correction dictionary, but also to the minimum frequency threshold for words when training the correction model for the entire data set, and to the similarity threshold for candidate evaluation in the correction workflow.
- We must consider lemmatization of words to further consolidate the vocabulary as well as removing stop words. Both the *spellux* package and the language support for Luxembourgish in *spaCy* have inbuilt options for lemmatization and stop word removal. The content analysis, however, shows that in some cases stop words (remember the example *eis Sprooch* “our language”) are part of discursive patterns that can be meaningfully interpreted.

### Measuring Attitudes Quantitatively Using a Mobile Crowdsourcing App

In the Schnëssen app, we use a classical questionnaire survey for data collection, in which the answers of the respondents are quantified using scaling. Compared to qualitative studies that work with interviews or ethnographic methods, this approach has the advantage of an easier evaluation and generalizability of the data. Results do not have to be condensed qualitatively based on categories derived from the data. Conversely, quantitative methods are not suitable for all aspects of attitudes research (see Casper, 2002 for a discussion), especially assuming that attitudes are situated evaluation routines that arise and come into play in practice (Purschke, 2015). For example, the complex Luxembourg multilingualism is not only organized according to social domains, which are relatively easy to query in a questionnaire study. In addition, the daily organization of language practice is highly dependent on individual factors, for example, the language skills of interlocuters, the social environment, and everyday routines, that influence the language preferences and the situational choice of a language. These can hardly be recorded using a general quantitative questionnaire.

Nevertheless, there are societal macro-conditions that lead to many people having comparable experiences that are anchored in their everyday social practice. This concerns, for example, language teaching in schools, which is partly responsible for the current poor image of French in the country, since the language is taught in a very formal and norm-oriented manner. The same applies to the country's global socio-economic demographic development that affects the language regime as a whole and that is being negotiated in public discourse, as can be seen from the RTL comments. Therefore, the questions in the questionnaire focus primarily on such aspects. In this way, we can ensure that the respondents already have the attitudes to be surveyed at their disposal because they are part of their everyday life experience.

The type of data collection using crowdsourcing also plays an important role in the composition and analysis of the data (see Entringer et al., forthcoming for a discussion). In principle, app-based crowdsourcing of linguistic data enables the collection of a large data set with comparatively little effort. However, we have to invest a lot of work in social media activities and public outreach in order to acquire enough respondents and to motivate them to a continued participation in longer survey campaigns. One technical challenge of the data set stems from the difficulties with matching social profiles and questionnaires. As a result, some of the completed questionnaires could not be considered for the analysis. However, on the basis of this identification, we can also compare the results of the questionnaire with the actual language use of the same participants in the app's recording task, for example, with regard to their attitudes toward German and French and their individual choice between competing lexical or phonological variants that originate from German or French. With regard to the demographic bias of the data basis, a targeted expansion of the sample by foreign residents and cross-border commuters would be desirable to get a more differentiated and comprehensive view of existing attitudes. To do this, we must also consider translating the questionnaire into other languages.

### Limitations of the Approach and Implications for Attitudes Research

The comparison of results using complementary data sets has proven to be insightful. For many questions from the questionnaire, we find corroborating evidence in the aggregated comment data. However, this this does not apply to all contexts. To illustrate this, we use one last question complex asking the participants about their attitudinal horizon for writing Luxembourgish (Table 10).

**Table 10:** Writing practice in Luxembourgish | *N* = 1,828, *p* < 0.001 (χ^2^).

	**Agree (%)**	**Somewhat yes (%)**	**Neither nor (%)**	**Somewhat no (%)**	**Disagree (%)**
“I do write texts in Luxembourgish in everyday life”	72.9	19.1	2.1	5.0	0.9
“I will write more texts in Luxembourgish in the future”	40.7	19.8	31.2	6.5	1.9
“When writing Luxembourgish, I should stick to the official rules”	37.9	41.3	10.8	8.1	1.9

The first question is an example that can be easily substantiated with the comment data even without querying the model. A large majority of respondents say that they write texts in Luxembourgish in everyday life, and this is exactly what the authors of the comments on RTL.lu do. The second question, on the other hand, cannot be easily converted into an informative query: the combination of s*chreiwen* (“to write”) and *Zukunft* (“future”) yields exclusively related verb concepts; the combination of *schreiwen, Zukunft*, and *Lëtzebuergesch* results mostly in related language concepts. Additionally, the third question documents potential discrepancies between the two data sets. While the majority of those questioned in the Schnëssen survey express a normative orientation toward the official spelling rules, the extent of orthographic variation in the comments proves the lack of practical implementation of these spelling rules. In view of the ongoing standardization of Luxembourgish, we can assume that the attitudinal orientation toward the norm precedes the actual practical acquisition of writing skills.

For the contrastive study of language attitudes, these findings mean that extensive contextual knowledge of the sociocultural, linguistic, and language-political context may be necessary to relate the results of the different analyses to one another in a meaningful way. At the same time, we can use this approach to investigate attitudes comprehensively (i.e., through complementary evidence from different datasets) and differentiated (e.g., regarding the difference between stances in discourse and connected underlying attitudes). Taken together, the results open up interesting perspectives both for attitudes research and for a culturally aware computational processing of text data. One particular challenge for further research in this context is the direct implementation of quantitative attitudes data in the training of word embedding models as a form of *social retrofitting* of such models.

## Conclusion

The aim of the present study was the contrasting investigation of language attitudes using the example of free text data from user comments and quantitative attitudes data from a survey. We have shown that sociolinguistic and computational methods can be successfully combined for the analysis of societal issues. This is confirmed by the correspondences between the attitudes reconstructed from the aggregated text data and the attitudes surveyed with the questionnaire. The results testify to the differentiated attitudinal horizons of the Luxembourgers concerning multilingualism in general and the individual languages in the language regime. The study also demonstrates the potential of computational sociolinguistics, at the center of which is the analysis of language as a sociocultural phenomenon. However, the work with the different approaches and data types also shows that we cannot interpret the results of the analysis without contextual knowledge about the sociolinguistic situation and the structure and dynamics of public discourse. Only the comparative analysis and embedding of the results in the larger sociocultural context allows us to make reliable statements about the research question at hand. It has also become clear that computational sociolinguistics needs a solid linguistic-theoretical basis and standardized technical-methodological procedures in order to fully unfold its potential for the study of language as a cultural phenomenon.

## Data Availability Statement

The datasets generated and analyzed for this study can be found on Zenodo: Luxembourgish word embedding model (user comments from RTL.lu): doi: 10.5281/zenodo.3978066; Schnëssen attitudes survey data: doi: 10.5281/zenodo.3978084.

## Ethics Statement

This research is in line with the rules and regulations for research ethics at the University of Luxembourg as stated in the official Ethics Review Committee policy (adopted by the Board of Governors at its meeting of October 25, 2019). The survey data from the Schnëssen project were collected on the basis of informed consent and were strictly anonymized for storage, processing, and analysis. The text data from the RTL news platform were provided by RTL in anonymous form. Identification of individuals based on the available data was not possible at any time.

## Author Contributions

All contributions (analyses, code, text) were made by CP. The data sources for the analyses were developed, collected, and prepared in collaboration with the colleagues from the projects Schnëssen and STRIPS.

## Conflict of Interest

The author declares that the research was conducted in the absence of any commercial or financial relationships that could be construed as a potential conflict of interest.

## Footnotes

^1^Detection accuracy was tested manually using a random sample of 1,000 texts labeled as Luxembourgish (100% correctly identified). Identification of non-Luxembourgish texts gives mixed results: Overall, accuracy is 64% for a random sample of 1,000 texts. Texts with wrong labels mainly concern very short texts that do not contain much language-specific content, or texts with a lot of code-switching. If we only consider texts with a length of more than 200 characters, the recognition rate increases to 96% for non-Luxembourgish texts.

^2^Language support for Luxembourgish in *spaCy* has been developed by the author and Peter Gilles.

^3^https://github.com/questoph/spellux/

^4^https://norvig.com/spell-correct.html

^5^See https://chd.lu, public petition No. 1404, for further details. In Luxembourg, any resident can submit a public petition on the website of the parliament. Provided it gets enough support (the current threshold is at 4,500 signatures), it will be officially discussed in parliament.
